# Supplementary material for: Dietary Heat-Treatment Contaminants Exposure and Cancer: A Case Study from Turkey
Source: Foods. 2023 Jun 9;12(12):2320. doi: 10.3390/foods12122320 (PMC10297194; doi:10.3390/foods12122320)
Supplement: Supplementary file 1 [file foods-12-02320-s001.zip › foods-2434899-supplementary.pdf]

Dietary Heat-Treatment Contaminants Exposure and Cancer: A Case Study from Türkiye

Hilal Pekmezci [1,*] and Burhan Basaran[2]

[1] Department of Elderly Care, Health Care Services Vocational School, Recep Tayyip Erdogan University, Rize, Turkiye. hilal.pekmezci@erdogan.edu.tr ORCID ID: 0000-0003-2157-4014

[2] Department of Nutrition and Dietetics, Faculty of Health Recep Tayyip Erdogan University, Rize, Türkiye. burhan.basaran@erdogan.edu.tr, ORCID ID: 0000-0001-6506-6113

[*] Correspondence: hilal.pekmezci@erdogan.edu.tr

Supplementary Table 1. Definition of dependent and independent variables (Regression analysis).

Dependent variables					
Models	Model 1	Model 2	Model 3	Model 4	Model 5
	Other (0)	Other (0)	Other (0)	Other (0)	Other (0)
Types of cancer	Cancers in the respiratory system (1)	Cancers in the reproductive system (1)	Cancers in the gastrointestinal tract (1)	Cancers in the urinary system (1)	Cancers in other systems (1)
**Independent variables**					
A. Total (Food groups)				Meat (red)	
B. Consumption frequency (Food groups)				Meat (white)	
C. Portion amount (Food groups)				Meat (fish)	
D. Cooking method (Food groups)				French fries	
E. Consumption mode (Food groups)				Bread	
				Coffee (instant)	
F. Frequency of consumption (Total), Portion amount (Total), Cooking method n (Total), Consumption mode (Total)				Coffee (ready to drink)	
				Coffee (Turkish coffee)	
				Black tea	

[a] Reproductive system cancer types=Breast, uterus, ovary. [b] Gastrointestinal system cancer types=esophagus, stomach, colorectum, pancreas, liver. [c] Urinary system cancer types=Prostate, bladder, kidney. [d] Respiratory cancer types=Lung, larynx, oral cavity/pharyngeal. [e] Other system cancer types= Brain, thyroid, lymphatic malignancies, skin, oro and hypopharynx, hematology.
